# Conceptual Foundations of Systems Biology Explaining Complex Cardiac Diseases

**DOI:** 10.3390/healthcare5010010

**Published:** 2017-02-21

**Authors:** George E. Louridas, Katerina G. Lourida

**Affiliations:** Department of Cardiology, Aristotle University, Thessaloniki 54124, Greece; katerina.lourida@gmail.com

**Keywords:** systems biology, heart failure, coronary artery disease, complex diseases, constraints, emergence

## Abstract

Systems biology is an important concept that connects molecular biology and genomics with computing science, mathematics and engineering. An endeavor is made in this paper to associate basic conceptual ideas of systems biology with clinical medicine. Complex cardiac diseases are clinical phenotypes generated by integration of genetic, molecular and environmental factors. Basic concepts of systems biology like network construction, modular thinking, biological constraints (downward biological direction) and emergence (upward biological direction) could be applied to clinical medicine. Especially, in the field of cardiology, these concepts can be used to explain complex clinical cardiac phenotypes like chronic heart failure and coronary artery disease. Cardiac diseases are biological complex entities which like other biological phenomena can be explained by a systems biology approach. The above powerful biological tools of systems biology can explain robustness growth and stability during disease process from modulation to phenotype. The purpose of the present review paper is to implement systems biology strategy and incorporate some conceptual issues raised by this approach into the clinical field of complex cardiac diseases. Cardiac disease process and progression can be addressed by the holistic realistic approach of systems biology in order to define in better terms earlier diagnosis and more effective therapy.

## 1. Introduction

Chronic complex diseases are multifarious in origin with a variety of biologically culpable components and environmental factors being implicated. Complex diseases are clinically progressive through multiple interactions between the involved components and environmental parameters. Heart failure (HF) and coronary artery disease (CAD) are chronic complex cardiac diseases promoted by the integration of various genetic, molecular and environmental factors. HF and CAD are complex adaptive systems and should be approached with the holistic methodology of systems biology. Technological progress of computational methodologies has motivated scientists and clinical practitioners to address complex pathologies. The advances of systems biology in the field of cardiovascular systems and the build-up of vast numbers of biological data have increased accurate analysis and integration of accumulated data [1]. The gradual build up of new scientific knowledge in the field of cardiology together with the advances in medical technology increased the chances to acquire further related information from complex medical datasets. Large numbers of computational data can be uploaded and maintained on vast remote servers (cloud computing). With the valuable accumulation of data it is possible to retrieve relevant medical information when it is needed and integrate this across data groups. Systems biology is the science that deals with integration of biological components producing ”system” of interacting molecules, networks, modules and phenotypes. Wolkenhauer [2] defines systems biology as “an approach to understanding complex, non-linear spatio-temporal phenomena, across multiple levels of structural and functional organization”. The principal objective of systems biology is to comprehend the way that interactive biological components construct functional networks at the level of organism and disease [3]. In addition, systems biology is concerned with the “emergent” functional properties derived from the interplay between genes, molecules, cells and tissues (upward biological direction), and with the restrictions imposed by “constraints” (downward biological direction) which establish biological robustness. After the advances made in molecular understanding of complex diseases it is inevitable for a shift of perspective in medical thinking from genomics to clinical phenotypes. Therefore, the interdisciplinary application of systems biology concept is extended to the level of clinical medicine. In explaining complex diseases new expressions are emerging based on systems biology phraseology like “systems medicine” which stands for the medical application of systems biology and “in silico medicine” that represents the “in silico computational integration of patient-specific data” [4].

Classical medical thought cannot predict clinical progression of complex cardiac diseases on the onset of disease. In contrast, using systems biology approach, in some significant aspects of complex cardiac diseases, like clinical progression and follow up, predictability may be possible. It seems that a disease process has hierarchical construction and clinical progression similar to the evolution-adaptation path of the biological world. Therefore, the extension of systems biology concepts to the domain of clinical medicine is possible and appealing. In clinical medicine, we are obliged to consider diseases as multileveled entities where dynamic molecular components and environmental events are integrated. Only, through this understanding is it possible to integrate clinical and basic science research in order to improve our knowledge and practice in the field of clinical medicine. This way, the use of systems biology methodology can be advantageous in the clinical domain for the welfare of patients and community.

Medical practitioners with knowledge in molecular biology and appreciation of the concepts of systems biology may approach diseases from different angle and describe disease complexity with unfamiliar ways. Yet, with systems biology, still remain some restrictions in order to understand the nature of a disease or to explain progression to various clinical phenotypes which continuously are changing. These limitations are extended in two fields of knowledge: in the time resolution of the disease phenotype and in the spatial (extension in different parts of the body) resolution of the biological background connected to the disease. It is possible, under the conceptual structure of systems biology, to integrate established features of complexity, network structure and hierarchical build-up that characterize a disease. All these features that distinguish a disease are taken into consideration in order to search for organizing principles during progression of a disease in space and time [5].

The purpose of this review paper is to incorporate and apply systems biology conceptual issues into the clinical field of complex cardiac diseases. The integration of data from both those scientific fields, clinical cardiology and systems biology, probably will give a better perspective in explaining complexity and clinical progression. Emergent properties of complex diseases and constraints imposed for enhanced disease robustness usually are the result of regional biological and environmental interactions. Only with thorough examination of the biological and environmental interacting parameters as one entity, will it be possible to define the role of each factor separately. In complex cardiac diseases the emphasis given to individual patient should be holistic in nature and focused more to the interaction between intrinsic biological parameters and extrinsic environmental factors.

## 2. Philosophical Aspects of Systems Biology

The concept of complexity is implicated in many natural systems like those existing in biology, environment and medicine including human diseases. For centuries, the explanation of the complex biological phenomenon was based on the process of reduction of complex structures into smaller components. This approach was named “reductionism” and had an impact on conventional clinical medical thought. Reductionism has described and analyzed complex diseases in all steps of clinical enquiries, including prognosis, diagnosis, prevention and mode of treatment. Despite important breakthroughs in clinical medicine, by the reductionist methodology, there are still unanswered questions and limitations for common complex diseases. Ahn et al. [6] suggest that “there are limits to reductionism, and an alternative explanation must be sought to complement it” and that “the systems perspective appreciates the holistic and composite characteristics of a problem and evaluates the problem with the use of computational and mathematical tools”. The same authors in another article claim that reductionism “dividing a problem into its parts leads to loss of important information about the whole” and that reduction “disregards component-component interactions and the dynamics that result from them” [7]. Thus, “systems medicine explores medicine beyond linear relationships and single parameters” and “involves multiple parameters obtained across multiple time points and spatial conditions to achieve a holistic perspective of an individual” [7].

The changes in biological sciences and clinical medicine oblige us to question some of the facts of reductionism in explaining complex phenomena. The concept of reductionism was dominant for many decades as the philosophical carrier of biochemistry, molecular biology and in some extent was the basis for clinical medicine. Nevertheless, the discipline of systems biology has grown into a promising branch of knowledge that can explain complex biological phenomena from molecular and cellular level to the field of practicing medicine.

In clinical medicine, the concept of phenotype under systems biology approach is the holistic expression of combined molecular interactions, in all levels of clinical progression, as the disease is evolving in space (many organs or tissues) and time. The reductionist concept of gene-dependent biological theory and the direct interrelation between genes and phenotypes have been replaced by the recognition of biological complexity, epigenetic effects and environmental processes. It seems that “reductionism is being displaced by systems biology, which favors the study of integrated systems” and that “integrated systems acquire new, system-level properties” [8]. Krohs and Callebaut [9], regard that systems biology has three scientific roots: (1) biological cybernetics and systems theory, (2) classical molecular biology, and (3) omics-disciplines. They stand up for the importance of omics data as central informational source to understand the concept of systems biology. They describe systems biology “as a merger of modeling and strategies from data-poor fields with data from fields that are data-rich, but largely deficient in explanatory modeling” [9,10].

In complex diseases, the relationship or interconnection between biological components is characterized by the complexity of three basic characteristics: the structure made by biological components, the pattern of their interconnection, and the process leading to phenotype. This approach is related to the notion of “three perspectives of organization, structure, and process that provide an integrative conceptual framework for the understanding of biological life” [11].

The human body as part of the biological world is organized as a complex system assembled from metabolic networks, biochemical pathways, functional components or modules, and phenotypes or models. The term “functional module” is applied to a group of autonomous and interconnected biological components that also possesses a discrete function under physiological or pathological circumstances [12,13]. Integration of complex networks from various biological hierarchies—genome, proteome, transcriptome, metabolome, cellular molecular components—builds-up models or phenotypes capable of interpreting complex biological phenomena or disease states. The integration principle of biological data can be extended to clinical level in an effort to predict the holistic behavior of functional modules and models underlying the disease state. It is impossible to predict the disease behavior by simple analysis of the properties of separated components.

Joyner and Pedersen [14], suggest that understanding of a disease process is accomplished through the perspective of integrative physiology as opposed to reductionism or systems biology. They claim, also, that the term “systems biology” is “narrowly defined” and based in “lack of fluency” of concepts such as homeostasis, regulated systems and redundancy. In the same line of interest, Kuster et al. [15] argue that systems biology is not a separate discipline with “omics” the active domain, but should be functioning as part of integrative physiology. Greenhaff and Hargreaves [16] believe that systems biology “is rooted in processes operating at a cellular level” and argue the presence of “conceptual inconsistencies between the fields of systems biology and integrative physiology in the context of exercise science”. Joyner [17] argues that Neo-Darwinism addresses human disease as an “oversimplified genotype equals phenotype” concept and that systems biology is cell centric that tries to bring together the genotype with the apparent complexity of human disease.

Nevertheless, in contrast to physiology, systems biology approach is integrative and also involves the study of new concepts like robustness, constraints and emergent properties applied in each level of complexity of human disease that is out of the sphere of integrative physiology. Thus, it seems that systems biology is crucial to reconcile reductionism with biological complexity explaining genesis and progression of human disease. Integrative physiology is largely occupied with “how the organism is working” under disease^’^s stress while systems biology has the potential to explain the beginning, progression and modes of treatment of complex diseases like heart failure and atherosclerosis [18]. Complex diseases are self-organized nonlinear processes that can be realized as dissipative biological constructions functioning far from natural equilibrium. For the practicing clinician, physiology is important because it includes the concepts of homeostasis, regulated systems and redundancy [14]. However, from our perspective this knowledge is not addressed to the disease as a functional entity. In studying pathophysiology the physician is concerned with the way that physiology is altered during the disease process, but for the clinician the disease entity is approached better with systems biology concept of emerging properties and constraints application. The emerging biological properties in each level -from the molecule to phenotype- are related to disease progression. The reference to the emerging properties in each step of disease is based on the interaction of various networks and modules in cellular, supracellular and clinical level. The incorporation of the biological or clinical emergent properties in each level of the clinical progression would facilitate also the understanding of the current and prospective therapy. Complexity of a multifarious disease demonstrates a clinical behavior that cannot be anticipated only by the isolated function of the genetic or molecular constituents. Systems biology application to clinical cardiology was reinforced by growth of other scientific fields like nonlinear dynamics, computational capabilities and chaos theory [19].

## 3. Directions and Disciplines

**Figure 1:**
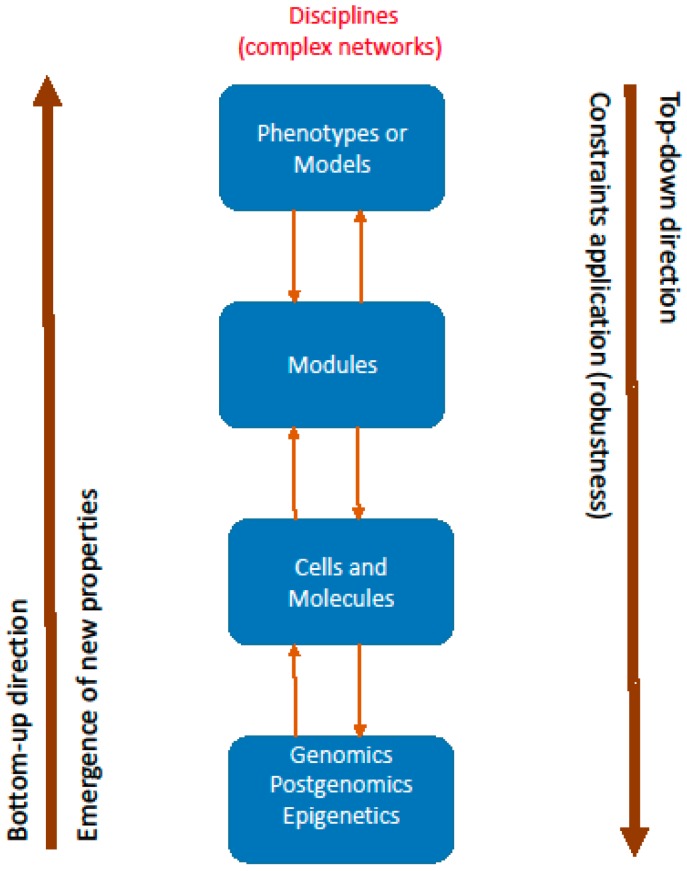
Concepts of systems biology: bottom-up and top-down directions; disciplines (complex networks); emergence of new properties; constraints application (robustness).

The accumulated disease-related data, network construction and clinical modeling, need the holistic approach of systems biology and a leveled enquiry to look into clinical progression. Two strategic directions for the study of complex diseases have been developed; the bottom-up (data about systems components) and the top-down (systems-level data) directions [9,10,18] (Figure 1).

The two strategies are complementary exploring the complexity that characterizes the hierarchical structure of cardiac diseases. It is acknowledged that in the bottom-up direction most important is the emergence of novel properties not expected from the isolated function of individual components. In addition, along the top-down direction significant is the construction of phenotypes (models), modules and networks that are underlying the disease state. The top-down direction is approaching “biological generalizations without the need for a full understanding of all molecular properties from the bottom-up” direction [10]. In other words we can describe phenotypes or modules or networks of a disease without fully knowing or understanding all the biochemical interconnections that are taking place. We could consider the disease entity as a complex whole of discrete biological mechanisms with some specific components and tasks that differ from the acknowledged normal components and functions. Furthermore, the disease entity is viewed as a layered biological construction with linked levels of “abnormal” molecular or clinical states.

**Figure 2:**
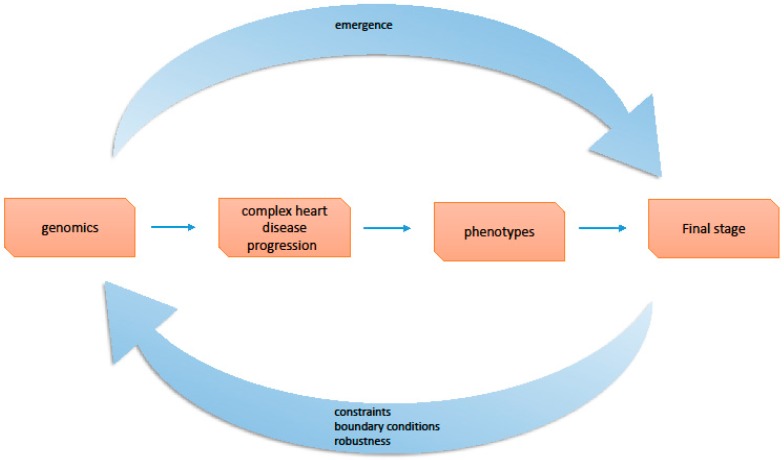
Progression of complex heart diseases: Relationship between emergent properties and constraints outlines progression of complex heart diseases.

The fields of molecular or clinical research are named disciplines as they are independent stages or levels of disease progression and each one of those is the object of intense research (Figure 2).

In HF, as disciplines are referred the levels (fields) of genomics and epigenetics, cellular networks, regulatory modules and clinical phenotypes. As modules in HF are referred, the regulatory mechanisms of natriuretic peptide system (NPS), sympathetic adrenergic system (SAS), renin-angiotensin-aldosterone system (RAAS) and left ventricular remodeling. In this paper, two clinical phenotypes of HF are mentioned: HF with preserved ejection fraction (HFpEF) and HF with reduced ejection fraction (HFrEF).

In CAD as disciplines are included the levels of genomics and epigenetics, cellular networks, coronary artery status and clinical phenotypes. The term of modules in CAD includes the coronary artery status like the pre-existent or post-acute artery obstruction, degree of coronary stenosis, coronary collaterals, myocardial or arterial remodeling and the environmental factors. Clinical phenotypes are the various appearances of CAD, like myocardial infarction, acute coronary syndromes and asymptomatic CAD [20].

## 4. Basic Concepts of Systems Biology Applied to Clinical Medicine

The most important concept of systems biology is the principle of robustness that stands for the presence of stability regardless of unsteady conditions [21]. Two other basic biological concepts are connected to systems biology robustness and are applied for the study of cardiac diseases; constraints and emergent properties. The above three adjacent concepts demonstrate strong causal connections between themselves and significant capacity of sequence. The emergent properties are upwardly causal (bottom-up direction) while constraints are downwardly causal (top-down direction) [22]. As they are applied in clinical medicine, the dialectic characteristics of the three basic biological concepts are able to elucidate contradictions and relations between levels of clinical complexity and figure out appropriate diagnostic or therapeutic solutions. Yet, many of the molecular components and networks participating in disease complexity remain undetermined.

### 4.1. Constraints

With systems biology approach, constraint-based explanation of the biological robustness is considered as a regulatory mechanism in metabolic networks [23,24]. Green and Jones [22], differentiated the mechanistic from the constraint-based explanations, and underlined that “while mechanistic explanations emphasize change-relating causal features, constraint-based explanations emphasize formal dependencies and generic organizational features that are relatively independent of lower-level changes in causal details”. In actual fact, the concept of constraints emphasizes the top-down causation from a higher level order to the pattern of processes at the lower levels [25]. The downward causation is the opposite of the reductionist principle because behavior of the biological elements of the lower level is determined by the behavior of the higher leveled order. Constraints are restricting some behaviors of the lower level while at the same time they allow other behaviors to appear [26]. The mathematical expression of the physical constraints as boundary conditions can be transferred to clinical cardiology thinking. This concept is extended from higher levels of clinical phenotypes and modules to lower levels of genes and proteins (Figure 1). The concepts of clinical boundaries or constraints could be used in clinical medicine and through this dialectic approach practicing physicians or cardiologists learn to react positively to the presence of a disease (Figure 2).

**Figure 3:**
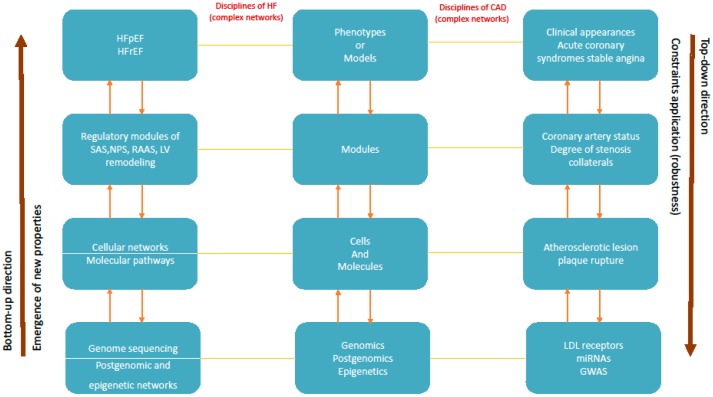
Progression of coronary artery disease and heart failure from systems biology perspective: application of systems biology concepts in HF and CAD progression. HF =heart failure; HFpEF = heart failure with preserved ejection fraction; HFrEF = heart failure with reduced ejection fraction; SAS = sympathetic adrenergic system; NPS = natriuretic peptide system; RAAS = renin -angiotensin- aldosterone system; LV = left ventricle; CAD = coronary artery disease; LDL = low density lipoprotein; miRNAs = microribonucleic acids; GWAS = genome wide association studies.

In medical terms this concept demonstrates how clinical constraints are imposed by phenotypes to lower level of modules. Clinical constraints, actually, decrease some degrees of freedom of the disease system enforced from phenotypes to lower level of modules and various networks. The above idea of constraint-based reasoning can be used to comprehend the interdependency between levels and explain complex disease progression. Complex cardiac diseases demonstrate significant robustness through functional enforcement of constraint-based relationships. This clinical robustness is analogous to the biological robustness, introduced by Kitano, as “a capacity for a biological system to maintain its performance across a range of perturbations to internal and external conditions” [21]. In practical terms this reasoning elucidates the dynamic functional interrelationship between disease levels, important for clinical decisions in diagnosis and therapy. For example, the clinical phenotypes of HF include many physiological regulatory systems (modules); most important are the neurohumoral and the cardiac remodeling systems [18]. Together with the function of the heart, regulatory systems are compensatory and significant for the homeostatic regulation of the body in an integrated functional network that includes molecular systems and organs. The failing myocardium increases activation of vasoconstrictive and vasodilatory compensatory systems in order to preserve cardiac output. These compensatory regulatory mechanisms belong to a lower level of activity compared to phenotypes and are regulated (constrained) by the degree of myocardium failure. Thus, the degree of modular activation is imposed and determined (constrained) with top-down causation by the higher level of phenotype according to compensatory needs of the failing myocardium (Figure 3).

In addition, the location and “the size of a myocardial infarction (phenotype higher level) will impose the extent of the post-infarction collaterals and the size of the myocardial remodeling (modular lower level)” [20]. This reasoning will influence clinical decision for medical or invasive therapy (Figure 3).

### 4.2. Emergence

The emergent functional characteristics originate from a self-organized process of biological components structured in a “system” with hierarchical order and bottom-up causation. The universal phenomenon of emergence is extended to molecular and clinical medicine, and implicates pathology and progression of complex diseases. Systems biology approach of emergence, applied as systems medicine at the clinical level, improves our understanding for the sequence of molecular and biochemical events that take place in cardiac diseases. This approach facilitates also the practicing clinician to comprehend why clinical picture progressively is changing, as disease is adjusted to novel emergent properties. The hierarchical network and modular construction, in a ladder of biological progression, from genes to phenotypes, involves emergence of new properties in each step of the biological process. The emergent new properties generate malfunctioning biological processes which participate in disease progression. This concept of emergence was developed in biology in order to understand and explain the phenomenon of life [27,28]. In the past, many articles have been published describing the emergent biological status and its characteristics [29,30]. It seems that the phenomenon of emergence depends on multiple factors interacting non-linearly, while the emergent properties in many ways are unexpected and possess level characteristics. Furthermore, functional emergent properties in each level which are provoked by non-linear interaction of multiple parameters, lead to functional connection (relationship) between successive biological levels. The emergent properties in each higher level are related or generated from the underlying processes of the lower level. This way, the specific structure of each lower level will have some connection to the emergent properties of the next higher level but this relationship is not completely causal. It seems that other factors are required, not necessarily directly causal, to explain differentiation and independence of emergent properties at the higher level from properties of the lower level. Cardiac diseases are organized in a complex functional manner with capacity of adaptation to a variety of internal and external environments.

The interaction of elements in each level with internal (comorbidities) and external environmental factors probably can explain partly the phenomenon of higher level emergence. However, current state of knowledge does not allow complete comprehension of the interconnection and interdependence between higher emergent properties and fundamental properties of lower level. In the realm of clinical medicine emergence of new properties in each level of complex cardiac diseases, is significant in order to explain the relentless progression of the disease. Emergence of new properties in cardiac diseases includes clinical signs and symptoms as well as worsening of the clinical status. Adaptation to noxious factors produces novel and irreducible emergent properties which cannot be explained through the reductionist integrative physiology of causal connection.

For example, in people with coronary atherosclerosis the size of atherosclerotic lesion and the degree of luminal artery obstruction (modules) are not implying that a myocardial infarction (phenotype) will definitely develop (Figure 3). The majority of coronary obstructions are not followed by myocardial infarctions but a lot of people are asymptomatic without ischemia or develop silent myocardial ischemia or angina. For a myocardial infarction to develop other internal and/or external environmental factors are obviously required [20].

## 5. From Networks to Modules and Models (Phenotypes)

The diverse and disparate field of “omics” and other biological datasets can be connected with well recognized biological pathways related to complex diseases. Metabolic interconnections are significant for many reasons; to locate important biomarkers or make a breakthrough on drug discovery or most important to recognize pathways related to clinical progression from molecules to phenotypes. In each discipline level, complex biological phenomena are better explained and understood by the conception of biological networks. Integration of disparate biological components in interacting biological networks is fundamental to understand cellular functions. Networks are composed of nodes (biological units) and edges or links (interactions between units), and produce a pattern of interconnections between components. Network behavior depends from the function of three factors: context (the components participating in a specific process), time (changing characteristics of each component), and space (topographic relationship between components) [6]. Many cellular networks-metabolic, signaling, and regulatory-have been described [31]. Aon [32], suggests that in view that “there is no direct relationship between metabolite, mRNA, protein, and gene” and in “order to be able to capture or explain developmental programs or the underlying mechanisms of a disease” it is essential to address the problem of biological interrelations with the network construction. He addresses the problem with the integration of “three different kinds of networks, mass-energy, information, and signaling” [32].

Analysis and evaluation of biological networks connected to human diseases give rise to the discipline of network medicine and network cardiology [33]. Application of the network concept to the domain of clinical cardiology produces interacting networks at the level of modules and phenotypes. The impact of network thinking in clinical cardiology is crucial in order to understand and describe the progressive nature of HF and CAD. Implication of the interactive networking system, to the final construction of modules and models, is changing the way we understand genesis and clinical deterioration of chronic heart diseases [34]. This approach explains the accumulated complexity of the biological phenomenon of the disease as it is progressing hierarchically from networks to modules and models integrating various unrelated biological compounds to the hierarchical system. At the present time of knowledge, all intermolecular links are not known across disciplines of complexity.

The main question in this inquiry is to recognize emerging principles and properties during shifting from one discipline to the next. Functional organization of biological systems is such that cooperation between interrelated components in networks and modules produce higher organized systems like disease phenotypes. Hierarchically structured disease conceptualization and the emergent clinical picture (like signs and symptoms) at the level of the phenotype, both increase our understanding of disease process. In biological networks there are non-random interactions (edges) between components (nodes) but there is intense clustering of adjacent nodes and long-distance linkage [10,35]. Barabasi and Oltvai [35] suggest that scale-free networks characterized by intense local nodal linkage and increased perceptibility of network properties are common in biological systems. In the realm of medicine these networks with high connectivity are basic structural elements for build-up of robust hierarchical systems during the disease process. Advance from biochemical pathways and networking to clinical modeling, is based on abstract thinking, but this type of reasoning is appropriate in order to unravel clinical progression. Today with advances in the field of molecular medicine and accumulated information on network construction, underling cellular and tissue functions, we can make this significant step from network thinking to clinical phenotypes.

Modules are critical functional entities which have a decisive impact on the structure of the model. Modules are networks with adaptive design characteristics that emerge spontaneously by self-organization. They are considered constructions of a higher level of complexity and participate in the progressive process of chronic diseases. Mathematical modeling of networking is examined extensively in different branches of science and medicine. In fact, we cannot interpret complex biological systems without modeling. In the past, modeling as a mathematical method was applied to the field of biological complexity and used for exploration of some basic concepts of the cardiovascular system. Modulation of cardiac action potential supported by Hodgkin-Huxley equations and mitochondrial ATP production are two examples of mathematical modeling application in the cardiovascular system [36,37]. At a clinical level, there are many functioning modules participating in construction of phenotypes. Some of the modules can change their function according to clinical set-up without disturbing the function of other modules. However, at the same time they are cooperating into new functions and contributing to disease’s clinical manifestations or progression.

Phenotype is a biological entity that could be applied to the level of molecules or networks or to the level of the clinical stage, and it encompasses a group of characteristics that could easily be identified as such. In the present paper, the term phenotype or model is addressed only to the clinical level and describes “appearances” of a disease process with clinically distinct or meaningful pathophysiological characteristics [38]. The term “clinical phenotype” is a mode of expression that incorporates anatomic, physiologic, biochemical and genetic parameters as well as clinical behavioral traits and complex interactions with a variety of intrinsic and extrinsic environmental factors [39]. Clinical phenotypic endurance to genetic, molecular or environmental perturbations characterizes the robustness of the disease phenotype. This phenotypic stability points to favorable adaptation to disturbed health equilibrium while phenotype’s robustness and behavior are controlled by constraints imposed by downward causation. However, when these perturbations exceed an upper limit of resistance then robustness is interrupted, with amendment of the phenotype features.

## 6. Repercussions to Clinical Cardiology

### 6.1. Personalized Medicine

Classical description of cardiac diseases in medical literature and guidelines are marked by their limited conception of disease genesis, progression and personalized treatment. With systems biology approach disease management or therapy is individualized with the complacent thought of “the most appropriate therapy should be adjusted to the patient’s needs” [40]. Personalized medicine is the application, to individual patient, of the holistic methodology of systems biology for a tailored and unique medical care. It is based on the integration of accumulated data from molecular stores, sequencing genomes, bioinformatics and biological or clinical networks [40]. In addition, biological and clinical networks integrate and interpret data from transcriptome, metabolome, proteome, comorbidities, environmental parameters and nutrition peculiarities. Patient-centered health policies are using all these variables in an attempt to improve follow-up of cardiac diseases from genesis to progression and phenotype construction.

The medical practitioner should address the patient as a person and not only inquire about clinical parameters or symptoms. For example, the experienced cardiologist should not address only angina as a symptom in patients with CAD, but, also, inquire about the patients’ whereabouts, personal or family medical history and personal preferences for the mode of treatment. All biological and clinical parameters involved in CAD like comorbidities, genetics and dietary practices, are integrated and conceived as a whole before prevention and therapy are undertaken. The patient is the primary object of interest having in mind that the exact pathogenic process underlying a complex cardiac disease is varied and disease phenotype is not always the same.

Systems biology uncovers biological pathways not previously comprehensible, identifies concealed biomarkers and generates novel therapeutics from isolated genes, metabolites and other biological substances. Personalized medicine is closely associated with applied methods for designing novel drugs. Systems approach, using molecular biomarker specific characteristics, improves “drug discovery and drug development process-encompassing a better molecular understanding of disease process, drug safety profiles and drug efficacy” [41]. The same authors claim that “novel therapies based on such molecular-system-based approaches are very appealing, but still they are in their infancy due to limited accessibility of robust and affordable molecular systems biology platforms” [41].

Ayers and Day [42] emphasize the idea that systems medicine “become one of the mainstays…of future research…not only for extracting further mechanistic knowledge…but also for faster and more effective drug development”. Connection of biological information with electronic medical records intends to revolutionize public health and clinical medicine with preventive and therapeutic programs more appealing to individual patient [43]. Translation of novel discoveries in the field of biomarkers and pharmacotherapy and their clinical implementation will eventually increase potential for personalized interventions. The Biochemical Pharmacology Discussion Group in a recent presentation described two major methods for designing pharmaceutical drugs; the traditional drug discovery (TDD) and the phenotypic drug discovery (PDD) [44]. The TDD method is “empiric design” and “researchers target a particular domain or protein after working to understand its mechanisms and molecular biology” while in PDD preferable method “many different compounds are tested on a system until one results in an observable phenotype of success, and the compounds’ mechanisms of action are not considered” [44].

There is a basic difference in clinical application of medical knowledge as it is extracted from various Practice Guidelines and as it is determined by the concept of Personalized Medicine. Guidelines are directed at populations and ignore pertinent factors related to individual patient like personal or family medical history and individual preferences towards disease management and therapy. Moreover, guidelines disregard differences in therapeutic effect of pharmacologic agents as well as interactions between drugs commonly seen in patients treated with multiple therapeutic medications. The concept of personalized medicine is based on the position taken by patient^’^s clinical practitioner who decides according to patient management inclination and disease variability and complexity [45].

### 6.2. Complex Cardiac Diseases

Systems biology application to clinical field, as systems medicine, is based to identification of important molecular and environmental interactions that affect genesis and progression of cardiac diseases. These interactions extend from intermolecular and network levels up to clinical level of modules and phenotypes. Clinical thinking in chronic diseases follows both directions of study, bottom-up and top-down, with interexchange of information between various stages (disciplines) of the disease (Figure 3). Cardiac complex diseases are multifaceted in origin with numerous variables, intrinsic (genetic, molecular, inflammatory) and extrinsic (environmental) to interact vigorously (Table 1).

The term “environmental” includes true external to the patient factors as well as comorbidities which participate in the demise of the patient. This unstable process is extended in time (chronic disease) and space (multi-organ process). Chronic and spatial variables are important not only for pathogenesis but also for disease progression.

### 6.3. Progression of Heart Failure

The assumption that the HF syndrome is a complex adaptive system could be used to understand the nature of HF clinical progression. The HF syndrome demonstrates a progressive clinical deterioration with characteristics of a dynamical and non-linear system with chaotic behavior [19]. The clinical deterioration of HF has the features of a complex and unstable system that is stabilized with a self-organized positive feedback neurohormonal and left ventricular remodeling mechanisms [19]. Therefore, the clinically progressive course of HF is determined decisively by the built-in mechanisms of neurohormonal compensatory regulatory systems and left ventricular remodeling compensation. HF syndrome is characterized by periods of clinical stabilization interrupted by periods of clinical instability. There is no effective therapy to strengthen stable equilibrium periods or to prevent instability and progression to final clinical stage [18]. Mann and Bristow [46] described the behavior of heart failure (HF) syndrome with systems biology approach and construction of clinical modules and models. They distinguished specific clinical syndromes or clinical models like cardiorenal model, cardiocirculatory or hemodynamic model, neurohormonal model and biomechanical model [46]. It is crucial to acknowledge that HF syndrome is a modular construction with predictable behavior of functional clinical models that involves biochemical networks, cellular and regulatory systems, and myocardial dysfunction [18,47]. The systems biology approach intends to identify molecular networks and regulatory systems that are linked to clinical properties. The role of individual genes in the pathogenesis and clinical progression of HF is limited to the field of hypertrophic and dilated cardiomyopathies which are caused by gene mutations. Hundreds of mutations were found in patients with hypertrophic cardiomyopathy responsible for different phenotypes. In general, some HF patients possess a complex multigenic inheritance but the significance of individual genes in pathogenesis and progression of HF is limited. In the HF process the role of transcriptomics, proteomics and metabolomics is increasing [18]. This enhances the possibility for early HF diagnosis with specific biomarkers and increases the chances of more precise treatment. However, in HF, the complete genetic network system and gene expression are still uncertain, and the whole of interactions between genomic, transcriptomic, proteomic and metabolomic systems are unknown. The metabolomic profile of patients with HF is based on circulating established biomarkers like the B-type natriuretic peptide (BNP) useful for diagnostic and prognostic reasons. Recently, attention was focused on the presence of various metabolites due to the diminished fatty acid oxidation, increased anaerobic glycolysis and ketone body oxidation, and impaired metabolism of branched-chain amino acids that occur in HF patients [48]. Thus, the HF process is complex and this, together with our fragmentary knowledge about the exact pathophysiology and progression, makes the creation of a complete HF network modeling difficult. The objective of the systems biology is the construction of novel networks, modules and models, and their integration with the existent ones. At the present time it is impossible to possess all the pertinent networks related to HF and merge them in a satisfactory clinical phenotype.

The clinical phenotype of patients with reduced ejection fraction (HFrEF) is extensively studied and understood but the clinical phenotype and natural history of patients with preserved ejection fraction (HFpEF) remain poorly defined [49]. The subclinical progression of the pre-clinical diastolic dysfunction (PDD) to the classical clinical phenotype of HFpEF is incompletely understood [49]. In HFrEF syndrome, systems biology approach explains better the importance of neurohormonal compensatory regulatory mechanisms like SAS, NPS and RAAS. The neurohormonal regulatory mechanisms are functional elements (modules) which together with left ventricular remodeling compensation are capable of stabilizing clinical progression, but not capable of preventing instability and progression to the last stage. Probably systems biology will provide more tools to understand the sequence of events in HFpEF and also to modify progression and final demise. The clinical understanding of HFpEF syndrome is increasing when examined with systems biology methodology, instead of the classical Oslerian method. Extensive prospective studies are required to comprehend the natural history of clinical progression in HFpEF patients and to determine the associated neurohormonal and left ventricular remodeling mechanisms that are involved [49].

### 6.4. Progression of Coronary Artery Disease

Atherosclerosis is the main cause of the sub-clinical and clinical forms of CAD and cerebrovascular diseases [50]. A sigmoidal (S-shaped) curve of development depicts atherosclerotic plaque and CAD progression “with a slow initial growth period of 30–50 years, followed by a fast expanding asymptomatic period of 10 years, and eventually by a final period with clinical symptoms” [20,51]. Genome-wide association studies (GWAS) were used to study complex diseases like CAD in order to identify loci connected with a particular disease. Bjorkegren et al. [51] declare that systems genetics are “a complementary approach to unlocking the CAD heritability and etiology”. They mention that GWAS recognize 153 possible CAD loci with 46 of those having genome-wide significance. These loci collectively can explain only <10% of genetic variance of CAD while the remaining 90% of CAD heritability is related to environmental factors. Genetic and environmental factors are two independent entities contributing to disease [51]. Thus, genetic contribution through mutational changes in many genes involved in genesis and progression of atherosclerosis has limited significance. The CAD loci identified by the GWAS are mainly associated to the early atherosclerotic course rather than to the later phases of the atherosclerotic clinical disease phenotypes. The GWAS approach is not able to recognize and explain the pathological changes and clinical progression of CAD phenotypes [21]. To extract more information in order to evaluate the heritability of CAD by both common and rare variants and to assess more accurately the clinical utility of genetic risk scores, very large sample sizes in mega-biobanks of at least half a million participants are needed [52]. Atherosclerotic process is a complex phenomenon involving epigenetic adjustments which are adapted and programmed to various gene expressions. Feinberg and Fallin [53] described epigenetics as the “information transmitted during cell division other than DNA sequence per se”. Proposed examples included “(1) DNA methylation… (2) posttranslational modifications of nucleosome proteins… and (3) the density of nucleosomes…” [53]. Recently, epigenetic changes and DNA methylation developed into a new field exploring atherosclerotic processes [54]. It seems that both genetic and lifestyle risks are independently related to development of CAD. Epidemiological and clinical trials established that various risk factors like lipoproteins, smoking, hypertension, diabetes and unhealthy lifestyle behavior are associated to atherosclerotic plaque pathogenesis and gradual progression to atherosclerotic disease. In a recent study “after quantifying both genetic and lifestyle risk among 55,685 participants” it was found “that adherence to a healthy lifestyle was associated with a substantially reduced risk of coronary artery disease” [55].

The new discipline of proteomics is focused on the characteristics of proteins in all biological systems including cells and tissues and their participation in health and human diseases. The role of proteomics in human diseases is more complicated as the complexity involves genetic variants, changed proteomes and environmental factors. All these parameters are integrated and interacted with limited predictive capability for the natural history of clinical progression. There are wide applications of proteomics in cardiovascular diseases as they are the identification of circulating protein biomarkers and the recognition of diseases^’^ pathophysiological mechanisms and potential therapeutic targets [56]. The evolution of isoform proteomics has significant consequences for cardiovascular research and probably for clinical application. In cardiovascular diseases these isoforms are represented “with differential expression levels or patterns, localizations, interactions, and post-translational modification in different cell types and during disease progression”[56]. These isoform changes are described during disease progression in ischemic cardiomyopathy, dilated cardiomyopathy, aortic stenosis and hypertrophy [57,58].

Metabolomic technologies allow us to quantify specific metabolites that reflect disturbances of myocardial metabolism. The main methods that estimate simultaneously a vast number of metabolites are the nuclear magnetic resonance (NMR) spectroscopy and mass spectrometry (MS) [59]. Myocardial metabolism is altered in heart diseases like in atherosclerotic process, CAD and HF. In addition, diabetes and obesity that are comorbidities associated to heart diseases and affecting many organs are responsible for generalized metabolic disturbances. Measuring specific metabolites the diagnostic and prognostic picture of a heart disease is becoming more realistic and more manageable. Metabolic processes are supporting cellular functions and participate in the well being of cells and tissues. Significant primary cellular function is the generation of energy in the form of adenosine triphosphate (ATP) from metabolically active energy sources. In heart diseases, energy cellular changes contribute to the cardiovascular pathology. Ussher et al. [59] emphasize that “during cardiometabolic disease progression, metabolic pathways are often perturbed and lead to the accumulation or loss of various metabolites that can be detected in the circulation via metabolomic profiling”. In patients with myocardial ischemia during exercise stress testing reduction of some metabolites of tricarboxylic acid (TCA) cycle, due to decrease in oxidative metabolic activity, was observed [60]. In ischemic myocardium both fatty acid and glucose oxidation rates are significantly decreased. Those changes are provoking important reduction in the myocardial extraction rate of free fatty acids and they are increasing the myocardial lactate [61,62]. Metabolomic profiling is an important screening procedure in order to predict subclinical atherosclerosis and identify patients at risk for early CAD. A number of circulating metabolites like the circulating trimethylamine-N-oxide and lysophosphatidylcholines are considered potential biomarkers which increase the risk of cardiovascular incidents [63,64].

Research on atherosclerotic plaque rupture reveals cellular and molecular processes that are fundamental for atherosclerotic plaque progression, from fatty streaks to intermediate lesion, up to the stage of plaque rupture and clinically important artery obstructive lesions [65,66]. Complexity of the atherosclerotic process and progressive nature of the sub-clinical and clinical obstructive coronary disease are meticulously explained and validated within the framework of systems biology. Intraluminal rupture of a non-obstructive plaque can produce a thrombus with partial or complete artery blockage and appearance of an acute coronary artery syndrome. An unbroken plaque eventually will grow and progress to a lesion which can obstruct the arterial lumen and induce symptomatic coronary obstructive disease [67].

Integration and translation of recent discoveries in the field of cellular and molecular domains that are related to genesis and progression of atherosclerotic plaques can increase our understanding for sub-clinical and clinical appearances of CAD. Molecular advances have reiterated the importance of low density lipoprotein (LDL) receptor expression on the level of LDL concentrations. Rise of LDL receptor expression lessens LDL levels and diminishes genesis or progression of atherosclerotic plaques. More than 1000 mutations in gene encoding LDL receptor were reported in a database for familiar hypercholesterolemia patients [68]. Lately was identified a mutation in the gene encoding protein 6 of LDL receptor (LRP6) in a family with autosomal dominant premature CAD and metabolic syndrome [69]. Lipoproteins and metabolism are interconnected and lead to atherosclerosis. Accumulated knowledge of LDL and high density lipoprotein (HDL) metabolism contributes to discovery of inhibitors of HMG-CoA reductase, drugs that are familiar as statins [67].

Other fields of research have been developed in order to identify culprit atherosclerotic targets which are considered important to follow-up progression of atherosclerotic process and myocardial ischemia. Recent procedures in imaging and computing data analysis improved disease understanding. Frueh et al. [70], describe a strategy based in 3-D imaging and computational methods in order to identify endothelial cells of interest covering atherosclerotic plaques in relation to biomechanical factors like shear stress and wall stress. The above applied methodology aimed to identify new signaling pathways and their reaction to blood flow with intention to increase understanding of mechanotransduction on endothelial cells and expand therapeutic potential [70]. Integration of biological and biomechanical factors constructs functional networks significant for further understanding of genesis and progression of atherosclerosis to clinical phenotypes. In coronary arteries, there is significant link between concentration of circulating plasma LDL and turbulent flow in areas of bifurcations or curbs. It was found that “in a top-down direction, in areas of bifurcation or trifurcation, the wall shear stress (WSS) is changing endothelial cells’ genes expression, which, in a bottom-up direction, promotes arterial remodeling and atherosclerosis” [20,71,72].

In atherosclerotic plaque destabilization process, many complex cellular and molecular mechanisms are involved. These mechanisms are not fully comprehended and many of those are questionable for their significance [73]. Szostak et al. [74], in ApoE^−/−^ mouse aorta, constructed an “atherosclerotic plaque destabilization biological network model with the semi-automated curation pipeline, Biological Expression Language Information Extraction Workflow (BELIEF)”. They concluded that “network models combined with the network perturbation amplitude algorithm provide a sensitive, quantitative method to follow disease progression at the molecular level” in order to “investigate and quantify molecular mechanisms during plaque progression” [74]. However, biological network models (BNMs) responsible for atherosclerotic plaque destabilization and able to quantify significance of biological processes in CAD, are not yet feasible [74,75].

The importance of the immune system in response to myocardial ischemia is recently reviewed with emphasis to imaging of immune activity in organs implicated in CAD [76]. Nahrendorf et al. [76] believe that control of the immune system^’^s post-myocardial infarction activity could possibly diminish re-infarction probability and HF appearance. Positron emission tomography (PET) with the use of ^18^F-fluorodeoxyglucose (FDG) stored in metabolically active cells can be used to mark inflammatory networks involved in myocardium, vessels and other target tissues. This way, most probably, PET findings combined with magnetic resonance imaging (MRI) data could decode systemic inflammatory networks in atherosclerosis or CAD in a pre-clinical stage or after ischemic events [77].

In general and in contrast to reductionist approach, systems biology strategy is a scientific method demanding “foundational questions” in order to study complexity as “the relationship between the whole and its parts” and to pursue complex cardiac diseases [18,78].

## 7. Conclusions

Chronic cardiac diseases like HF and CAD are complex adaptive systems promoted by integration of various genetic, molecular and environmental factors and approached with the holistic methodology of systems biology. Human HF and CAD are considered as self-organized biological systems, progressive in nature, which could be explained successfully with systems biology approach. Systems biology methodology provides important tools for biological and clinical analysis of complexity which characterizes cardiac diseases. Systems biology approach implicates two directions of study, bottom-up (genes to phenotypes) and top-down (phenotypes to genes), and provides leveled understanding of disease progression. In addition, it is explaining the emergent (upward direction) functional properties of disease in each level of progression and describes the imposed constraints (downward direction) that establish robustness for modular stability and phenotype disease progression.

## Figures and Tables

**Table 1:** Clinical understanding of chronic cardiac diseases with reductionism and systems biology approach.

Medical Applications	Reductionism’s Objectives	Systems Biology Holistic Strategy
Clinical focus	Isolated clinical parameters	Interactions between components, like molecules, networks, modules, models (phenotypes)
Prevention	Isolated culprit molecular and environmental parameters	As an entity the whole range of culpable variables
Diagnosis	Isolated molecules, biomarkers, signs, symptoms	The patient as a “diseased person”
Therapy	Treating causes and symptoms	Treating the patient from an holistic perspective
